# Possible Influence of δ-Aminolevulinic Acid Dehydratase Polymorphism and Susceptibility to Renal Toxicity of Lead: A Study of a Vietnamese Population

**DOI:** 10.1289/ehp.7904

**Published:** 2005-06-01

**Authors:** Sin Eng Chia, Huijun Zhou, Mei Theng Tham, Eric Yap, Nguyen-Viet Dong, NguyenThi Hong Tu, Kee Seng Chia

**Affiliations:** 1 Department of Community, Occupational and Family Medicine, National University of Singapore, Singapore, Republic of Singapore; 2 Center of Occupational Health and Environment, Ministry of Industry, Hanoi, Vietnam; 3 General Department of Preventive Medicine and Control of HIV/AIDS Control, Ministry of Health, Hanoi, Vietnam

**Keywords:** δ-aminolevulinic acid dehydratase (ALAD), *HpyCH4*, intron, lead, SNP (single-nucleotide polymorphism), urinary albumin (Ualb), urinary retinol-binding protein (URBP), urinary α_1_-microglobulin (Uα1m), urinary β_2_-microglobulin (Uβ2m)

## Abstract

We examined six newly identified polymorphisms in the δ-aminolevulinic acid dehydratase (*ALAD*) single-nucleotide polymorphisms (SNPs) to determine if these SNPs could modify the relationship between blood lead (PbB) and some renal parameters. This is a cross-sectional study of 276 lead-exposed workers in Vietnam. All workers were measured for PbB, urinary retinol-binding protein (URBP), urinary α_1_-microglobulin (Uα1m), urinary β_2_-microglobulin (Uβ2m), urinary *N*-acetyl-β-d-glucosaminidase (NAG), urinary aminolevulinic acid (ALAU), serum α_1_-microglobulin (Sα1m), serum β_2_-microglobulin (Sβ2m), and urinary albumin (Ualb). The six SNPs were *Msp* and *Rsa* in exon 4, *Rsa39488* in exon 5, *HpyIV* and *HpyCH4* in intron 6, and *Sau3A* in intron 12. Analysis of covariance (ANCOVA) with interaction of PbB × SNPs were applied to examine modifying effect of the SNPs on the association of renal parameters and PbB, adjusting for potential confounders of age, gender, body mass index, and exposure duration. *HpyCH4* was found to be associated with certain renal parameters. For *HpyCH4 1-1*, an increase of 1 μg/dL PbB caused an increase of 1.042 mg/g creatinine (Cr) Uα1m, 1.069 mg/g Cr Uβ2m, 1.038 mg/g Cr URBP, and 1.033 mg/g Cr Ualb, whereas in *HpyCH4 1-2*, an increase of 1 μg/dL PbB resulted in an increase of only 1.009 mg/g Cr Uα1m, 1.012 mg/g Cr Uβ2m, 1.009 mg/g Cr URBP, and 1.007 mg/g Cr Ualb. *HpyCH4* SNP appeared to modify the lead toxicity to kidney with wild-type allele being more susceptible than variants. The mechanism for this effect is not clear. Further studies are needed to confirm this observation.

The first and most common δ-aminolevulinic acid dehydratase (*ALAD*) polymorphism studied was the *Msp* single-nucleotide polymorphism (SNP) in exon 4. Since it was first reported (Battistuzzi et al. 1981), many reports (e.g., Onalaja and Claudio 2000) have been published on its association with inorganic lead (henceforth referred to as lead). Epidemiologic studies have tried to determine if the *Msp* polymorphism is important in human susceptibility to lead regarding various lead-targeted systems (Alexander et al. 1998; Bergdahl et al. 1997; Hu et al. 2001; Schwartz et al. 1995, 2000).

In recent years, reports have been published on the association between the Msp SNP and renal effects of lead exposure. Smith et al. (1995) suggested that the *Msp-2* variant is a more susceptible allele because *Msp-2* carriers had higher concentrations of blood uric acid and blood urea nitrogen (BUN). The differences achieved only borderline significance after adjusting for potential confounders. Bergdahl et al. (1997) found that serum creatinine is higher for *Msp-2* carriers than for *Msp 1-1* homozygotes in a sample of 89 lead workers. A linear relationship was observed between patella lead concentration, using X-ray fluorescence method, and serum uric acid when patellar lead passes 15 μg/g bone mineral for *Msp-2* carriers, but the similar linear relation can be seen only when patella lead was > 101 μg/g bone mineral for the *Msp 1-1* group (Wu et al. 2003). Wu et al. (2003) postulated that lead toxicity became apparent at a lower exposure level for *Msp-2* carriers than for *Msp 1-1* persons. These studies suggested that *Msp-2* carriers were more susceptible to lead effects than were *Msp 1-1* homozygotes. However, the reports were not consistent. Some studies had reported that the *Msp-2* variant was associated with higher creatinine clearance as well as lower mean serum creatinine and BUN (Weaver et al. 2003).

Renal parameters such as creatinine clearance, BUN, and serum creatinine are insensitive to renal function changes. As much as 50–70% of kidney nephrons must be damaged before variation can be detected (Goyer 1989). The consensus may not be so clear, however, regarding the effects of lead on the renal system because no good longitudinal studies have been conducted to establish the predictive value of early biologic exposure markers in lead-exposed workers. Roels et al. (1994) studied 76 male lead smelter workers and 68 controls matched for age, gender, socioeconomic state, residence, and work shift characteristics. Although the tibia bone lead, blood lead (PbB), and urinary lead levels of the exposure group were significantly higher than those of the control group, no significant differences were observed in either common and stimulated creatinine clearance. More recently, however, Weaver et al. (2003) reported that higher lead levels were associated with lower BUN and serum creatinine levels and higher calculated creatinine clearance among those with the *ALAD 1-2* genotype.

Renal parameters such as low-molecular-weight proteins [e.g., retinol-binding protein, α_1_-microglobulin, β_2_-microglobulin, *N*-acetyl-β-d-glucosaminidase (NAG)] representing proximal tubule injury are considered good alternatives because early lead-induced nephropathy usually involves damage of proximal tubule cell (Nolan and Shaikh 1992). To date, several studies have shown that these renal parameters have good correlation with lead exposure indices. NAG has been found to be the only marker elevated in early nephropathy in five studies reviewed by Bernard and Lauwerys (1989). One study in Japan (Endo et al. 1993) claimed that urinary α_1_-microglobulin (Uα1m) can be a useful indicator of renal impairment. A study of 128 lead workers (Chia et al. 1995) reported that Uα1m appears to be the most sensitive parameter compared with urinary β_2_-microglobulin (Uβ2m) and urinary retinol-binding protein (URBP). Another study of environmental lead exposure in children showed that URBP is increased by lead with good dose–effect and dose–response relations with PbB (Bernard et al. 1995).

To date, 111 SNPs have been reported in the National Center for Biotechnology Information website (NCBI 2005). But when we assessed the website in June 2004, 46 SNPs were reported. What are the relationships of these 46 SNPs with the renal effect of lead exposure? We used the Helix Tree software (Golden Helix, Inc., Bozeman, MT, USA; http://www.goldenhelix.com/pharmhelixtreefeatures.html) to test for linkage disequilibrium among the 46 SNPs; 6 SNPs were shown not to be in linkage disequilibrium. In this study we examined these 6 SNPs (*Msp* and *Rsa* in exon 4, *Rsa39488* in exon 5, *HpyIV* and *HpyCH4* in intron 6, *Sau3A* in intron 12) and examined their association with certain renal functions among a group of lead-exposed workers in Vietnam.

## Materials and Methods

### Study population.

The study population consisted of 323 workers from a battery factory in Hai Phong City, Vietnam. All workers from the production line as well as in the division of management and quality control were recruited into this study. Of these 323 workers, 246 were occupationally exposed to lead, and the remaining 77 were not directly exposed to lead. Although these 77 workers were not directly exposed to lead, many had a previous history of exposure. Some were still exposed, albeit less than the 246 group. Therefore, these workers were also included in the study. Signed consent was obtained for each worker before blood and urine samples were taken for subsequent analysis. The workers also filled out a questionnaire (in Vietnamese) with the help of a Vietnamese interviewer. After completing the questionnaire, a spot urine sample and 10 mL of blood were collected from each worker during the medical examination. Twenty of the exposed lead workers were not present during the study period, and 17 did not want to participate. Of the 77 workers who were not directly exposed to lead, 10 were unwilling to give their urine and blood for analysis. Because the study was strictly on a voluntary basis, the workers’ decisions in not giving urine and/or blood were respected. Hence, the 47 workers were excluded in the study, giving a response rate of 88.2% (276 of 323).

### Questionnaire.

Information gathered included age, years of education, detailed occupational history, and current and previous smoking habits. Alcohol intake was also documented carefully, as it could confound the findings. Actual amount of alcohol consumption per day (i.e., types of drink consumed and the amount estimated by number of bottles consumed) and number of years of drinking were noted. Six workers gave a history of diabetes mellitus and/or hypertension. These workers were excluded from the study because diabetes mellitus and hypertension are known to affect renal functions.

### Laboratory analysis.

We obtained blood samples by venipuncture with lead-free disposable syringes and stored the samples in heparinized lead-free polypropylene tubes. PbB was analyzed using atomic absorption spectrophotometry (Varian Spectra AA-30; SiberHegner Pte Ltd., Victoria, Australia) with a graphite furnace. External quality control was performed yearly under the National External Quality Assurance Scheme (England) and Inter-laboratory Comparison Programme (Canada). We obtained spot urine samples, and those used for analysis of ALAU, URBP, Uα1m, Uβ2m, urinary albumin (Ualb), and NAG were buffered (pH 7.2). Serum and urine samples were stored at –30°C until analysis. We analyzed all samples within 1 month of arrival in Singapore at our laboratory and adjusted all urine parameters for variability in urine flow using urinary creatinine that was expressed per gram of creatinine. Urine creatinine concentrations were determined using standard laboratory techniques. Ualb, ALAU, URBP, Uα1m, Uβ2m, and NAG were measured by enzyme-linked immunosorbent assay using commercially available polyclonal antibodies or test kits (Roche Diagnostic Corp., Indianapolis, IN, USA). The details of these tests have been reported previously (Green et al. 2004). NAG was determined using Noto’s method (Chia et al. 1995)

### Identification of ALAD polymorphisms.

Six polymorphisms located on the *ALAD* gene were selected for analyses in our study. They are *MspI* and *RsaI* polymorphisms, which span a 248-bp region of the genomic *ALAD* sequence, as well as *Rsa39488* (232 bp), *HpyIV* (234 bp), *HpyCH4* (213 bp), and *Sau3AI* (282 bp) polymorphisms. The products of interest were determined through amplification by polymerase chain reaction (PCR), using primers designed with Primer3 (Whitehead Institute for Biomedical Research, Cambridge, MA, USA; http://frodo.wi.mit.edu/cgi-bin/primer3/primer3_www.cgi). The amplification cycles were performed on the PTC-100 MJ Thermal Cycler (PerkinElmer, Inc., Boston, MA, USA) and optimized to ensure specific amplification of the products of interest. Each PCR reaction was performed in a final volume of 25 μL, which included 10 ng of pure genomic DNA, 1.0 μM each primer, 200 μM each dNTP, 1× Taq buffer, 1.5 mM MgCl_2_, and 1.0 U of Taq DNA polymerase (all from Promega Corp., Madison, WI, USA), and topped up to the required volume with sterile distilled water. We used the restriction fragment length polymorphism method to locate the site of mutation; the fragments were determined on a 2.0% agarose gel (Seakem agarose; Cambrex North Brunswick Inc., North Brunswick, NJ, USA) and visualized on the Biorad Gel Doc 2000 system(BioRad Laboratories, Hercules, CA, USA).

### Statistical analysis.

We determined that 28 people did not have any genotype measurements and had to exclude them from the analysis, leaving a sample of 248 workers. The data set of these 248 workers was used in all subsequent analyses. For some of the biologic samples, we were not able to analyze all the parameters because these samples ran out before we could complete the test.

The distributions of variables were checked individually with histogram and one-sample Kolmogorov-Smirnov (K-S) test for normality. Necessary transformation was undertaken to achieve normal distribution for all important variables. In the end, ALAU, Uα1m, Uβ2m, Ualb, URBP, serum α_1_-microglobulin (Sα1m), and serum β_2_-microglobulin (Sβ2m) went through logarithmic transformation. NAG went through square root transformation. The transformed variables all follow normal distribution with an insignificant *p*-value from the one-sample K-S test.

Statistical analysis addressed two issues: *a*) Is there any difference in the mean PbB concentrations and renal parameters between genotypes of each SNP after adjusting for known confounders? *b*) Within each single SNP, is the effect of PbB concentration on the renal parameters similar across genotype subgroups? Analysis of covariance (ANCOVA; Kutner et al. 2003) was the main statistical technique used. We initially examined scatter plots of each renal parameter (*y*-axis transformed value) and PbB concentration (*x*-axis) to identify linearity and extreme points.

**Figure 1:**
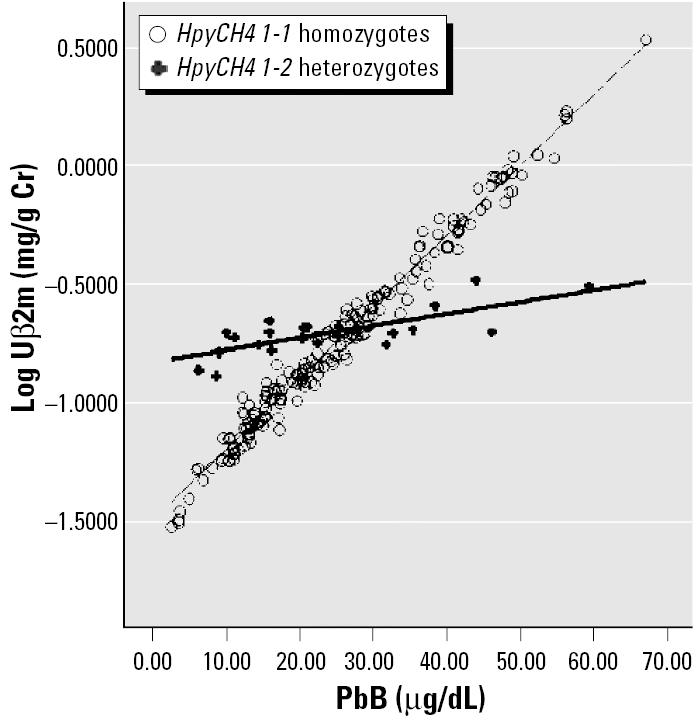
Regression lines of Uβ2m versus PbB by *HpyCH4* genotype, adjusted for age, exposure duration, gender, and BMI. Data points are specified by value pairs of blood lead (PbB) and log-transformed renal parameters (Uα1m, Uβ2m, URBP, Ualb).

**Figure 2:**
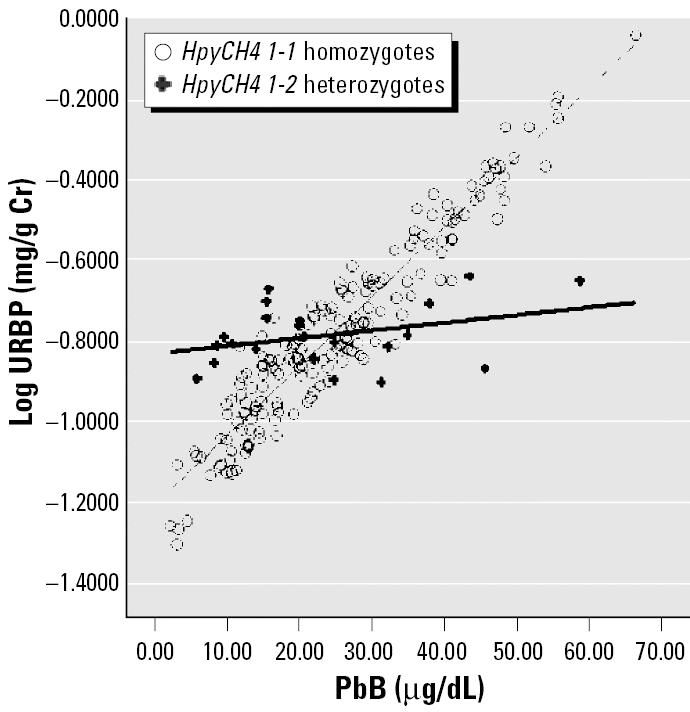
Regression lines of URBP versus PbB by *HpyCH4* genotype adjusted for age, exposure duration, gender, and BMI.

**Figure 3:**
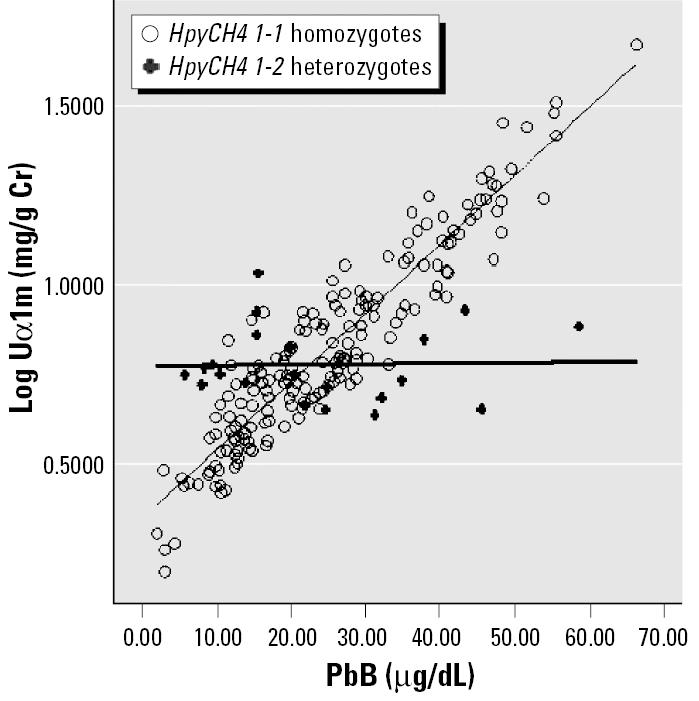
Regression lines of Uα1m versus PbB by *HpyCH4* genotype adjusted for age, exposure duration, gender, and BMI.

**Figure 4:**
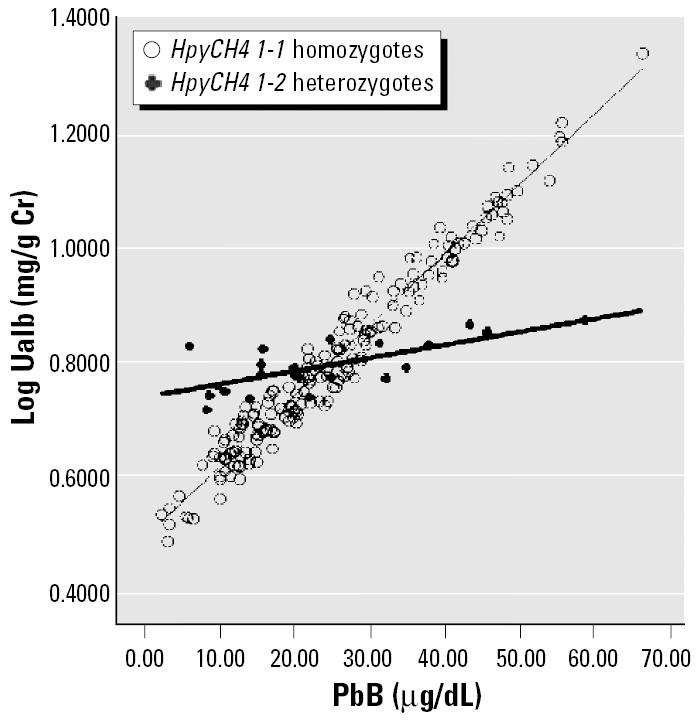
Regression lines of Ualb versus PbB by *HpyCH4* genotype adjusted for age, exposure duration, gender, and BMI.

We addressed the first issue using ANCOVA to adjust for age, exposure duration, body mass index (BMI), PbB (where appropriate), and gender. For the second issue, an interaction term of PbB × SNP was included while adjusting for the known confounders. Multiply linear regression equation models were used in the generation of regressions lines for Figures 1–4. All data are processed by SPSS (version 12.0; SPSS Inc., Chicago, IL, USA). Significance level was set as *p* < 0.05 (two-sided).

## Results

Of the 248 workers, 184 (74.2%) were male and 64 (25.8%) were female, with a mean age of 39 (range, 20–66) years. The range of exposure duration was 1–41 years. Demographic features by SNP are summarized in Table 1. The allelic and genotypic frequencies are summarized in Table 2. For *Msp*, *Sau3A*, *HpyIV*, and *HpyCH4*, the wild-type allele is more predominant, whereas for *Rsa* and *Rsa39488*, the variant allele is more prevalent. The basic characteristics of the measurement variables are shown in Table 3. Table 4 shows the comparison of mean renal parameters by SNPs. The *HpyIV* variant group had higher mean ALAU and Ualb levels than the group with the wild-type genotype (1.15 vs. 0.95 mg/g Cr and 7.75 vs. 7.37 mg/g Cr, respectively). The wild-type genotype group for the *Sau3A* marker (*1-1*) had lower ALAU levels compared with those of the variant group (0.93 vs. 1.08 mg/g Cr) but higher NAG concentrations (2.93 vs. 2.53 U/g Cr). Only *Rsa39488* subgroups had significant differences in PbB, with means of *ALAD 1-1*, *ALAD 1-2*, and *ALAD 2-2* being 21.87 μg/dL, 20.43 μg/dL, and 25.54 μg/dL, respectively. The group with the *Rsa39488* variant genotype, *2-2*, had much higher PbB levels than the *1-1* and *1-2* genotype groups (Table 4).

Multiple linear regression equation models were constructed for each of the six SNPs. Of these six SNPs, only HpyCH4 showed significant association with URBP, Uα1m, and Uβ2m in the multiple regression analysis. Table 5 shows the relationship between different renal parameters and the HpyCH4 SNP. PbB concentrations significantly affect URBP, Uα1m, Uβ2m, and Ualb even after adjusting for age, gender, BMI, and exposure duration. PbB is an important predictor for these renal indices. There are significant interactions between the HpyCH4 marker and PbB concentration (Table 5). The associations of PbB concentrations and renal parameters are presented in Figures 1–4.

The increment in renal parameters corresponding to unit increase in PbB concentration is higher for HpyCH4 1-1 homozygotes than for HpyCH4 1-2 workers. For Uα1m, each increase of 1 μg/dL PbB corresponds to an increase of 0.016 log Uα1m (anti-log 1.042 mg/g Cr) for HpyCH4 1-1 workers, whereas for HpyCH4 1-2 workers the increase is 0.004 (anti-log 1.009 mg/g Cr). The same trend is shown in the relations of Uβ2m, URBP, and Ualb to PbB concentrations. The contrast between HpyCH4 1-1 and HpyCH4 1-2 is 1.069 versus 1.012 mg/g Cr, 1.038 versus 1.009 mg/g Cr, and 1.033 versus 1.007 mg/g Cr for Uβ2m, URBP, and Ualb, respectively.

## Discussion

For ALAD SNPs, this study is the first to address more than one SNP. In addition to Msp in exon 4, we also examined Rsa SNP in exon 4, Rsa39488 in exon 5, HpyCH4 and HpyIV in intron 6, and Sau3A in intron 12. It has been shown by linkage analyses that these six SNPs are not in linkage disequilibrium.

We did not find any correlation of lead with Sα1m or Sβ2m, which is consistent with other studies (Cardenas et al. 1993; Chia et al. 1994; Endo et al. 1993). In our study, Msp SNP in exon 4 constitutes 95.9% of ALAD1 and 4.1% of ALAD2 alleles. This pattern is similar to those in most studies conducted on Asian populations (Hsieh et al. 2000; Shen et al. 2001). The allele composition of the other five SNPs had not been reported. To our knowledge, this is the first report of genetic distribution of the ALAD polymorphism in a Vietnamese population.

Although we determined some positive findings with Rsa, Rsa39488, HpyIV, and Sau3A, our findings were not consistent. Therefore, these SNPs are not discussed further; we concentrate our discussion on HpyCH4 and its association with renal function. HpyCH4 is a SNP that involves a G/C transversion in gene position 12,916 in intron 6. This SNP was first reported by Olson (2000). Allele frequency in his sample (North American population) was 0.818 for HpyCH4-1 and 0.182 for HpyCH4-2, and genotype frequency is 0.727 for HpyCH4 1-1, 0.182 for HpyCH4 1-2, and 0.091 for HpyCH4 2-2. Nakamura (2002) reported the frequencies of HpyCH4-1 to be 0.808 and of HpyCH4-2 to be 0.191 in a sample of 734 Japanese. In our study, HpyCH4-1 has a frequency of 0.942, and HpyCH4-2 has a frequency of 0.058. HpyCH4 1-1 homozygotes accounted for 88.4% (191), HpyCH4 1-2 heterozygotes accounted for 11.6% (25). Variant HpyCH4 2-2 homozygotes were not detected in this study population. Our study frequencies of the HpyCH4 allele were fairly similar to those reported by Nakamura (2002), whose sample was also drawn from an Asian population. However, the proportion of HpyCH4-1 in our study was relatively higher.

Several studies have shown that URBP (Bernard et al. 1995), Uα1m (Chia et al. 1995; Endo et al. 1993), Uβ2m (Nolan and Shaikh 1992), and urinary NAG (Bernard and Lauwerys 1989) are good indicators of early renal effects due to lead exposure. A study of 128 lead workers in Singapore (Chia et al. 1995) showed that Uα1m appears to be the most sensitive parameter compared with Uβ2m and URBP.

Our study found that a newly identified ALAD polymorphism, HpyCH4 in intron 6, was able to modify the association of PbB concentrations with certain renal parameters. For HpyCH4 1-1 homozygotes, 1 μg/dL PbB caused an increase of 1.042 mg/g Cr Uα1m, 1.069 mg/g Cr Ub2m, 1.038 mg/g Cr URBP, and 1.033 mg/g Cr Ualb, whereas in HpyCH4 1-2 heterozygotes, an increase of 1 μg/dL PbB resulted in an increase of only 1.009 mg/g Cr Uα1m, 1.012 mg/g Cr Uβ2m, 1.009 mg/g Cr URBP, and 1.007 mg/g Cr Ualb. These findings suggest that the HpyCH4 1-2 variant could be more resistant to the effects of lead toxicity on some renal functions than HpyCH4 1-1.

We showed that in the relationship between PbB and some renal parameters (Figures 1–4), two regression lines (HpyCH4 1-1 and HpyCH4 1-2) intersect around 25 μg/dL PbB. This point highlights the importance of stratifying the data by PbB when we study the modification effects of ALAD polymorphism on associations between lead exposure and renal function. Otherwise, problems would arise and cause bias in the findings. In our study, for workers with PbB < 25 μg/dL, HpyCH4 2-2 workers have higher renal function values than HpyCH4 1-1 workers. The opposite is true for workers with PbB > 25 μg/dL. Thus, one may conclude that ALAD2 is more susceptible when the samples have low lead exposure. Conversely, one might find that ALAD1 is more susceptible when the samples have higher exposure (i.e., PbB > 25 μg/dL). Our findings may explain, to some degree, why there were contradicting reports on the association between PbB concentrations and ALAD alleles in some studies. Wetmur et al. (1991) found a significant over-representation of Msp-2 isozymes among individuals with PbB in excess of 30 μg/dL. Schwartz et al. (1995) reported that the over-representation of Msp-2 allele can be present only when the PbB was > 40 μg/dL. Smith et al. (1995) failed to show any association of Msp-2 to PbB and he ascribed the nonassociation with the low lead exposure of the study population (mean PbB, 7.78 μg/dL).

For the Msp polymorphism, the differences noted in relation to the susceptibility of effects of lead have been attributed to the differential binding abilities of the ALAD isozymes. However, the HpyCH4 SNP, located in the intron region of the ALAD gene, technically does not have any protein-coding functions involved. George and David (1998) observed that some DNA behaves as an exon when expressed by one pathway but as an intron when expressed by another pathway. Both pathways can operate simultaneously, resulting in greater protein product variety. Perhaps the ALAD enzyme encoded by HpyCH4 1-1 and HpyCH4 1-2 could have been produced through one such pathway (George and David 1998). Regulatory functions of introns may involve controlling gene activity in different developmental stages or responding to immediate biologic needs by controlling local gene expressions. This function of introns could occur if exons code for a domain, a polypeptide unit that has a discrete function such as binding to a membrane, the catalytic site of an enzyme, or a structural unit of a protein (Jerry 2001). However, few studies have reported that polymorphisms located in introns can be associated with lead toxicity. One study that examined Msp polymorphism in intron 2 reported that variant workers were associated with decreased bone lead but not PbB (Kamel et al. 2003). Recent work has demonstrated that intronic mutations can have functional consequences in some genes such as p53 (Lehman et al. 2000). Similarly, intronic mutations may also have functional consequences in the ALAD genes.

Some limitations are inherent in this study. It is possible that individuals with certain ALAD polymorphisms may be more susceptible to the effects of lead toxicity on the renal system and thus would have been excluded from the workforce even before this study was conducted (healthy worker effect). But these susceptible workers will need to have obvious clinical renal diseases to be removed from the workforce. This is first time that factory workers in Vietnam were tested with these renal tests. Therefore, it is highly unlikely that we are dealing with a healthy worker effect. In Vietnam, most factories are state owned, as is this factory. Workers work in a factory all their lives (as can be seen by the long exposure history in Table 1). Schwartz et al. (1995) reported differences in the Msp ALAD genotype frequencies among workers in three lead factories with different lead exposures. They postulated that workers with the more susceptible ALAD genotype may moved to a lower lead-exposed job. Conversely, workers with the protective ALAD genotype may continue working because they have fewer symptoms even in higher exposure areas. We are unable to study this possible “population stratification” bias because our workers were rotated through the different departments/sections in the factory depending on the job schedule and manpower requirement.

We did not measure the body burden of lead and thus could not examine the effects of the ALAD SNPs in relation to lead accumulation in the body. We did use exposure duration as a surrogate, and this factor has been adjusted for in our analysis. In spite of factoring in the duration of exposure to lead, workers with HpyCH4 1-2 genotype had significantly better renal (certain) parameters than workers with the HpyCH4 1-1 genotype. The sample size of our study is also not large. There were only 25 workers with HpyCH4 1-2 compared with 191 workers with HpyCH4 1-1. Cadmium is also known to affect some of the renal parameters that were measured in this study (Chia et al. 1989). It has been reported that urinary cadmium of 4 μg/g Cr is the critical value for cadmium to have a significant effect on Uα1m, Uβ2m, URBP, and NAG (Roels et al. 1993). There was no measurement of cadmium levels for all the workers. However, we randomly selected a sample of 25 workers and determined their urine cadmium level; the mean was 0.57 μg/g Cr with a range of 0.34–0.98 μg/g Cr. These values were far below the reported urinary cadmium of 4 μg/g Cr that would affect some of the studied renal parameters.

In conclusion, *HpyCH4* SNP located in intron 6 may have modifying effects on the relationship between lead exposure and renal function, with individuals carrying the *HpyCH4 1-1* genotype being more susceptible to lead toxicity of the kidneys. The mechanism for this is unclear. Further studies are needed to confirm this observation.

## Figures and Tables

**Table 1:** Demographic characteristics of the study population by SNPs.

		Gender		Age (years)		Exposure (years)		
SNP	Genotype	Male [*n* (%)]	Female [*n* (%)]	*n* (%)	Mean ± SD	*n*	Mean ± SD	Range
*Msp*	*1-1*	168 (92.8)	58 (92.1)	226 (92.6)	39.5 ± 10.6	214	15.4 ± 10.2	1–41
	*1-2/2-2*	13 (7.2)	5 (7.9)	18 (7.4)	36.4 ± 9.8	18	10.8 ± 9.1	1–27
*Rsa*	*1-1*	40 (22.1)	16 (26.2)	56 (23.1)	39.8 ± 11.0	54	15.8 ± 11.2	1–41
	*1-2*	86 (47.5)	28 (45.9)	114 (47.1)	38.4 ± 10.3	107	14.3 ± 9.9	1–39
	*2-2*	55 (30.4)	17 (27.9)	72 (29.8)	39.7 ± 10.7	69	15.0 ± 9.9	1–36
*Rsa39488*	*1-1*	41 (23.0)	15 (23.8)	56 (23.2)	40.0 ± 11.0	55	16.0 ± 11.3	1–41
	*1-2*	81 (45.5)	32 (50.8)	113 (46.9)	39.3 ± 10.2	105	14.9 ± 10.0	1–39
	*2-2*	56 (31.5)	16 (25.4)	72 (29.9)	39.0 ± 10.7	69	14.5 ± 9.5	1–36
*HpyCH4*	*1-1*	136 (87.2)	55 (91.7)	191 (88.4)	39.2 ± 10.4	184	15.5 ± 10.2	1–41
	*1-2*	20 (12.8)	5 (8.3)	25 (11.6)	40.9 ± 10.7	23	13.9 ± 9.3	1–30
*HpyIV*	*1-1*	131 (74.9)	42 (67.7)	173 (73.0)	39.8 ± 10.4	163	15.3 ± 10.2	1–41
	*1-2/2-2*	44 (25.1)	20 (32.3)	64 (27.0)	38.1 ± 10.9	62	14.4 ± 10.3	1–39
*Sau3A*	*1-1*	106 (66.7)	31 (51.7)	137 (62.6)	38.3* ± 10.7	131	14.9 ± 10.1	1–39
	1-2/2-2	53 (33.3)	29 (48.3)	82 (37.4)	41.4* ± 9.7	77	16.05 ± 10.0	1–41

*p < 0.05.

**Table 2:** Frequencies of alleles and genotypes for each SNP [% (n)].

SNP	ALAD1	ALAD2	1-1	1-2	2-2
Msp	95.9	4.1	92.6 (226)	6.6 (16)	0.8 (2)
Rsa	46.7	53.3	23.1 (56)	47.1 (114)	29.8 (72)
Rsa39488	46.7	53.3	23.2 (56)	46.9 (113)	29.9 (72)
HpyCH4	94.2	5.8	88.4 (191)	11.6 (25)	0
HpyIV	85.2	14.8	73 (173)	24.5 (58)	2.5 (6)
Sau3A	76.9	23.1	62.6 (137)	28.8 (63)	8.7 (19)

The distribution of genotypes and alleles within each SNP has been proven to follow the Hardy-Weinberg equilibrium.

**Table 3:** Mean of exposure indices and renal parameters.

Biologic parameter	n^a^	Mean ± SD	Minimum–maximum	Range
PbB (μg/dL)^b^	247	24.4 ± 13.6	2.0–66.9	64.9
ALAU (mg/g Cr)	236	0.95 ± 1.55	0.34–3.63	3.30
Sα1m (mg/L)	241	41.8 ± 1.29	20.84–74.25	53.42
Sβ2m (mg/L)	244	1.45 ± 1.31	0.77–2.85	2.09
NAG (U/g Cr)^c^	236	2.82 ± 0.18	0.10–7.60	7.50
Uα1m (mg/g Cr)	244	6.82 ± 1.91	1.29–27.32	26.03
Ualb (mg/g Cr)	239	7.06 ± 1.73	1.23–34.12	32.89
Uβ2m (mg/g Cr)	237	0.19 ± 2.30	0.02–1.81	1.79
URBP (mg/g Cr)	239	0.16 ± 1.95	0.03–0.86	0.83

Cr, creatinine. Data are geometric mean, except as indicated.

a Numbers differ between tests because some samples were too small for all the tests to be performed.

b Arithmetic mean.

c Square root mean.

**Table 4:** Mean exposure indices and renal parameters by SNP genotypes.

	HpyCH4		HpyIV		Msp		Rsa			Rsa39488			Sau3A		
Adjusted mean^a^	1-1	1-2	1-1	1-2/2-2	1-1	1-2/2-2	1-1	1-2	2-2	1-1	1-2	2-2	1-1	1-2/2-2
PbB (μg/dL)^b^	22.68	17.89	23.32	20.52	22.68	17.89	21.13	21.89	24.22	21.87*	20.43*	25.54*	23.22	20.8
ALAU (mg/g Cr)	1	1	0.954**	1.150**	1.01	0.95	1.07	0.98	0.99	1.03	1.02	0.96	0.93**	1.08**
Sα1m (mg/L)	42.02	43.9	42.32	42.02	42.21	40.97	42.23	41.99	42.24	42.29	42.04	42.58	42.04	42.6
Sβ2m (mg/L)	1.41	1.43	1.4	1.4	1.41	1.49	1.36	1.43	1.4	1.37	1.42	1.46	1.4	1.39
NAG (U/g Cr)^c^	2.81	2.46	2.78	2.77	2.77	2.76	3.12	2.73	2.66	3.07	2.81	2.51	2.93*	2.53*
URBP (mg/g Cr)	0.15	0.13	0.15	0.15	0.15	0.14	0.16	0.14	0.14	0.16	0.14	0.14	0.15	0.14
Uβ2m (mg/g Cr)	0.18	0.21	0.18	0.19	0.18	0.24	0.18	0.19	0.18	0.18	0.17	0.2	0.18	0.18
Ualb (mg/g Cr)	7.35	6.92	7.374*	7.750*	7.29	7.19	7.37	6.84	8.1	7.55	6.98	7.79	7.6	6.79
Uα1m (mg/g Cr)	6.21	5.29	6.15	6.07	6.11	6.8	6.49	5.69	6.58	6.5	5.72	6.53	5.89	6.36

Data are geometric means adjusted for age, exposure duration, gender, BMI, and PbB, unless otherwise specified.

a Adjusted for age, exposure duration, gender, and BMI.

b Arithmetic mean.

c Square root mean.

*p < 0.05.

**p < 0.01.

**Table 5:** Models of HpyCH4 regression by renal parameters.

Variable	β(95% CI)	p-Values
Log URBP		
R2 = 0.1473		
PbB	0.016 (0.006, 0.027)	0.002
HpyCH4	–0.02 (–0.140, 0.100)	0.742
HpyCH4 × PbB	–0.012 (–0.022, –0.003)	0.008
Log Uα1m		
R2 = 0.2366		
PbB	0.018 (0.008, 0.027)	< 0.001
HpyCH4	–0.067 (–0.177, 0.043)	0.233
HpyCH4 × PbB	–0.014 (–0.022, –0.005)	0.002
Log Uβ2m		
R2 = 0.1417		
PbB	0.029 (0.016, 0.042)	< 0.001
HpyCH4	0.047 (–0.103, 0.197)	0.537
HpyCH4 × PbB	–0.024 (–0.035, –0.012)	< 0.001
Log Ualb		
R2 = 0.1417		
PbB	0.014 (0.005, 0.023)	0.002
HpyCH4	0.000 (–0.103, 0.103)	0.998
HpyCH4 × PbB	–0.011 (–0.019, –0.003)	0.007

CI, confidence interval.

The model adjusts for age, gender, BMI, and exposure duration. 0 represents the HpyCH4 1-1; 1 represents HpyCH4 1-2. The reference group is HpyCH4 1-1; the β-coefficient of PbB is the slope for the association between PbB and renal parameters in participants with this genotype. The corresponding slope in workers with ALAD 1-2 is the sum of the β-coefficient of PbB and that of HpyCH4 × PbB in each model (i.e., in the URBP model, the β-coefficient of PbB for HpyCH4 1-1 group is 0.016; the corresponding βfor HpyCH4 1-2 is 0.004 = 0.016 + –0.012). p-Values for the HpyCH4 × PbB reflect the statistical significance of the difference between the slopes of the regression line for HpyCH4 1-1 and for HpyCH4 1-2 workers.
